# Second opinion machine learning for fast-track pathway assignment in hip and knee replacement surgery: the use of patient-reported outcome measures

**DOI:** 10.1186/s12911-024-02602-3

**Published:** 2024-07-23

**Authors:** Andrea Campagner, Frida Milella, Giuseppe Banfi, Federico Cabitza

**Affiliations:** 1 IRCCS Ospedale Galeazzi Sant’Ambrogio, Milan, Italy; 2 Department of Computer Science, Systems and Communication, University of Milano-Bicocca, Milan, Italy; 3 Faculty of Medicine and Surgery, Universitá Vita-Salute San Raffaele, Milan, Italy

**Keywords:** Medical machine learning, Patient-reported outcome measures, Second opinion, Fast track, Controllable AI, Medical decision making

## Abstract

**Background:**

The frequency of hip and knee arthroplasty surgeries has been rising steadily in recent decades. This trend is attributed to an aging population, leading to increased demands on healthcare systems. Fast Track (FT) surgical protocols, perioperative procedures designed to expedite patient recovery and early mobilization, have demonstrated efficacy in reducing hospital stays, convalescence periods, and associated costs. However, the criteria for selecting patients for FT procedures have not fully capitalized on the available patient data, including patient-reported outcome measures (PROMs).

**Methods:**

Our study focused on developing machine learning (ML) models to support decision making in assigning patients to FT procedures, utilizing data from patients’ self-reported health status. These models are specifically designed to predict the potential health status improvement in patients initially selected for FT. Our approach focused on techniques inspired by the concept of controllable AI. This includes eXplainable AI (XAI), which aims to make the model’s recommendations comprehensible to clinicians, and cautious prediction, a method used to alert clinicians about potential control losses, thereby enhancing the models’ trustworthiness and reliability.

**Results:**

Our models were trained and tested using a dataset comprising 899 records from individual patients admitted to the FT program at IRCCS Ospedale Galeazzi-Sant’Ambrogio. After training and selecting hyper-parameters, the models were assessed using a separate internal test set. The interpretable models demonstrated performance on par or even better than the most effective ‘black-box’ model (Random Forest). These models achieved sensitivity, specificity, and positive predictive value (PPV) exceeding 70%, with an area under the curve (AUC) greater than 80%. The cautious prediction models exhibited enhanced performance while maintaining satisfactory coverage (over 50%). Further, when externally validated on a separate cohort from the same hospital-comprising patients from a subsequent time period-the models showed no pragmatically notable decline in performance.

**Conclusions:**

Our results demonstrate the effectiveness of utilizing PROMs as basis to develop ML models for planning assignments to FT procedures. Notably, the application of controllable AI techniques, particularly those based on XAI and cautious prediction, emerges as a promising approach. These techniques provide reliable and interpretable support, essential for informed decision-making in clinical processes.

## Introduction

In medical practice, seeking second opinions is a common and valued approach to achieve consensus in diagnosing and managing patient care, thereby enhancing the overall quality of healthcare [1]. This practice becomes particularly crucial in situations involving complex healthcare decisions, those that are potentially distressing for the patient, or when significant risks are involved [2]. Contrary to cases where patients themselves seek a second opinion for confirmation of a diagnosis or due to unsatisfactory interactions with their doctors, second opinions initiated by other parties, especially those initiated by doctors, often aim to restrict the use of low-value treatments (which are those offering minimal or no benefit, posing potential harm, or yielding marginal benefits at disproportionately high costs [3]). Therefore, within the realm of clinical decision making, a second opinion serves as a significant decision-support, which enables another physician to either confirm or alter the proposed treatment plan [4] and has been proven to significantly reduce medication errors [5], including diagnostic mistakes [2].

Machine Learning (ML) algorithms have increasingly been applied to augment clinical decision-making in recent years across various tasks [6, 7]. Particularly, to counteract cognitive biases associated with an over-reliance on decision support technologies, ML algorithms have recently been utilized as tools for offering second opinions [8, 9]. In this context, they are viewed as cognitive supports with specialized capacities, designed to confirm or revise (i.e., augment) decisions initially made by clinicians, rather than merely automating the clinical decision-making process [10]. Several studies have explored the impact of algorithmic assistance on clinicians’ diagnostic performance when supplemented by a second opinion from an ML algorithm. For example, Gurusamy et al. [11] investigated the use of ML models for providing second-opinion recommendations in brain tumor classification. Kovalenko et al. [12] developed a prototype ML-based video analytics system to aid in diagnosing Parkinson’s disease. Cabitza et al. [13] assessed various ML-based second opinion protocols to enhance the diagnostic accuracy of orthopedists in radiological knee lesion readings. Bennasar et al. [14] created an ML-based second opinion system for predicting root canal treatment outcomes. Similarly, Rosinski et al. [15] proposed an ML-based system for selecting assistive technology in post-stroke patients. While most of these studies primarily focused on the use of ML algorithms for second opinion support in diagnostic or prognostic tasks, our article shifts focus to another aspect of clinical process management - the assignment of rehabilitation protocols. Specifically, we develop second-opinion decision-support ML models for the assignment of patients to surgical Fast Track (FT) in hip and knee arthroplasty.

In the field of orthopedics, the FT surgical procedure represents a rapid rehabilitation protocol designed to mitigate the physiological and psychological stresses typically associated with surgery [16]. Its primary aim is to facilitate early mobilization and recovery post-surgery [16], leading to outcomes such as reduced Length Of hospital Stay (LOS) [17], decreased convalescence time [18], and lower overall costs [19]. However, the criteria for FT patient assignment have not fully leveraged the extensive patient data available, including Patient Reported Outcome Measures (PROMs). A few studies have focused on comparing the effectiveness of Fast Track versus Care-as-Usual surgical procedures from a patient-centered perspective (e.g., [20]). More generally, some studies have considered the application of ML in management of orthopedics’ patients [21], with a specific focus on the prediction of the length of stay [22, 23], which, similarly to FT can be useful to better manage bed availability as well as identifying patients who are most in need of increased rehabilitation theory. By contrast, to our knowledge, no study has yet developed second opinion ML models specifically for decision support in the assignment of patients to surgical FT: thus, the focus on this task represents a crucial element of novelty in our contribution.

To achieve our objective, we utilized ML models designed to predict whether a patient, preliminarily assigned to FT by their managing clinician, will experience an improvement in health status. In this context, improvement serves as a proxy for the effectiveness of the FT procedure. Accordingly, the model either validates the clinician’s decision to assign the patient to FT (if an improvement is predicted) or suggests a need to reconsider this assignment in favor of an alternative approach, such as Care-as-Usual, or prompts the managing clinicians to more thoroughly assess the patient’s specific situation. As these models provide second-opinion support for clinicians, we have developed them based on principles of *controllable AI* [24], ensuring that the support offered is comprehensible and, if necessary, rejected by the managing clinicians. In line with the definition by Kieseberg et al. [24], we define ‘controllable AI’ as second-opinion AI systems that are not only accurate but also capable of identifying and signaling *control loss* conditions, wherein the effectiveness cannot be fully assured, necessitating or warranting human intervention to evaluate the second opinion support. Our focus was particularly on methods for detecting control loss, i.e. situations of high uncertainty or potential anomalies, by using *eXplainable AI* (XAI) [25] to ensure model recommendations are understandable to clinicians, and *cautious prediction* [26] for uncertainty quantification and to enhance reliability.

## Methods

This retrospective study was conducted using a dataset derived from the electronic health records (EHRs) of IRCCS Ospedale Galeazzi - Sant’Ambrogio (OGSA), a leading orthopedic teaching and research hospital in Italy. Our focus was on developing a second-opinion model; therefore, we exclusively analyzed records of patients who were part of the perioperative Fast Track process. The dataset encompassed 925 individual patient records, collected over the period from January 2018 to November 2020.

The dataset for this study included a comprehensive range of patient data, covering demographic characteristics (such as Sex, Age, Weight, Height, and Body Mass Index [BMI]), details about the assigned surgical procedure and primary affected area (including ICD code, and distinction between Knee and Hip surgeries, as well as First intervention vs Revision), clinical information (ASA Class, Pre-surgery Hemoglobin levels), and preoperative PROMs scores (VAS, EQ5D, SF12 Mental score, SF12 Physical score). Additionally, the SF12 Physical score recorded at the 3-month follow-up was also included. The distribution of these features is detailed in Table 1.

**Table 1:** Table of descriptive statistics for data features with *P*-Value Analysis: The table presents the descriptive statistics for each feature in the dataset, stratified by ‘Improved’ and ‘Not Improved’ sub-cohorts

Feature	Mean (Not Impr.)	St.Dev (Not Impr.)	Mean (Impr.)	St.Dev. (Impr.)	Missing (Not Impr.)	Missing (Impr.)	*P*-value
Age	67.100	10.690	66.579	10.496	0%	0%	0.493
VAS (Preop)	6.856	2.333	7.226	2.031	0.4%	1.5%	0.069
SF12 Physical (Preop)	38.812	8.439	30.868	6.449	0%	0%	< 0.001*
SF12 Mental (Preop)	49.365	11.362	51.495	11.996	0%	0%	0.011*
EQ5D (Preop)	0.760	0.126	0.708	0.111	0%	0.3%	< 0.001*
Height	166.777	9.074	167.685	9.033	1.6%	0.7%	0.288
Weight	75.543	14.461	77.583	15.455	1.6%	0.7%	0.179
BMI	27.119	4.566	27.486	4.413	1.6%	0.7%	0.236
Hb (Preop)	13.922	1.352	14.066	1.399	0.8%	0.4%	0.248
Feature	Categories (Not Impr.)			Categories (Impr.)			*P*-value
Sex	Female (42.2%), Male (57.8%)			Female (48.4%), Male (51.6%)			0.229
Hip/Knee	Hip (42.2%), Knee (57.8%)			Hip (62.5%), Knee (37.5%)			< 0.001*
First Intervention	First Intervention (96%), Revision (4%)			First Intervention (96.3%), Revision (3.6%)			0.191
ASA	1 (11.6%), 2 (86.1%), 3 (2.3%)			1 (14.4%), 2 (81.6%), 3 (4%)			1 (0.443), 2 (0.162), 3 (0.159)

For continuous and ordinal features, differences were assessed using the Mann-Whitney U test, while for categorical features, the Fisher’s exact test was utilized to determine statistical significance

Asterisk denotes a significant difference between the two cohorts, at the 95% confidence level

As outlined in the Introduction section, our approach for providing second-opinion support involved using a proxy for the potential effectiveness of assigning patients to the Fast Track program, namely the improvement in the patients’ health status. Specifically, we defined the target variable as a binary outcome (Improved vs. Not Improved) determined by changes in the SF12 Physical score at the 3-month follow-up. A patient was classified as ‘Improved’ if the difference between their 3-month follow-up score and the preoperative score exceeded the distribution-based Minimum Clinically Important Difference (MCID) for this score [20]^1^. If this threshold was not met, patients were categorized as ‘Not Improved.

Due to the presence of records with missing values, we elected to exclude any patient records that lacked even one of the features under consideration. This resulted in the removal of incomplete data, leaving 899 records available for subsequent analysis. It is noteworthy that the distribution of the target variable was unbalanced: 644 patients (approximately 71.6%) were classified in the ‘Improved’ category, whereas 255 patients (about 28.4%) fell into the ‘Not Improved’ category. Apart from the one-hot encoding of categorical variables, no additional pre-processing procedures were undertaken: specifically, we did not implement any pre-processing method to correct the imbalance in label distribution.

In the development of ML models for this study, we considered a variety of model classes, encompassing both ‘black-box’ approaches known for their efficacy with tabular data [27], as well as models grounded in XAI principles. In alignment with the concepts of controllable AI outlined in the Introduction section, the XAI models were specifically chosen for their interpretability [28]. This feature enables clinicians to ‘look into the models’, thereby understanding the basis of the second-opinion support and potentially identifying classification errors. Among the black-box models, we included Random Forest (RF), Support Vector Machines (SVM), XGBoost (XGB) and Multi-layer Perceptron (MLP). Regarding XAI methods, we opted for Logistic Regression (LR) and Decision Tree (DT), along with two advanced, state-of-the-art approaches: Hierarchical Shrinked Trees (HST) [29] and Fast Interpretable Greedy-Tree Sums (FIGS) [30]. HST functions as a post-hoc regularization method to streamline decision tree models by shrinking predictions at each node towards the sample means of their ancestors. Conversely, FIGS represents a generalization of the CART algorithm, operating by constructing a forest of simple trees through a greedy approach based on boosting principles, with the trees being subsequently combined in summation.

All the models were trained with the objective of predicting the target variable, namely, classifying each patient as either ‘Improved’ or ‘Not Improved’, based on the aforementioned features. Prior to training, the dataset was divided into two distinct sets: a training set and a test set. This division followed a stratified split of 75% for training and 25% for testing. The training set was used for both the training of the models and the optimization of hyper-parameters. On the other hand, the test set was used for a blind evaluation of the results to assess the models’ performance. The test set size was selected based on a minimum sample size determination criterion, so as to ensure that with high probability (greater than 95%) the measured estimates of performance would be close to the true performance values.

The models were implemented as pipelined models encompassing three different steps: feature scaling, feature selection and predictive model. The full list of hyper-parameters for the three different steps of each pipeline model is reported in Table 2. In particular, we used a class weighting hyper-parameter to control label imbalance: we either considered equal weighting of the instances (i.e., ignoring label imbalance) or weighting more the instances in the negative class (i.e., label imbalance correction). All other hyper-parameters not specified in Table 2 were set to the default values, except for random seeds that were all set to the value 0 to ensure reproducibility. As mentioned above, training and hyper-parameter optimization was performed only on the training set, in order to avoid data leakage and overfitting, using a cross-validation approach. The training set was split into 5 folds (each of which encompassed 15% of the original dataset), and at each iteration 4 folds (60% of the original dataset) were used for training and hyper-parameter selection, while the remaining fold was used for internal evaluation. The performance of each model and hyper-parameter configuration was determined as the average of the reported performance across the five iterations of the cross-validation and measured in terms of the Balanced Accuracy, so as to account for the label imbalance. For each model, we selected the configuration of hyper-parameters that reported the best performance on the cross-validation and then re-trained the model on the entire training set.

**Table 2:** Hyper-parameters for the developed models

Hyper-parameter name	Range	Selected value
Scaling Method	min-max, standard, Yeo-Johnson, max-abs, normalize, robust, None	LR: max-abs; DT: robust; SVM: min-max; RF: Yeo-Johnson; XGB: min-max, MLP: max-abs, FIGS: None, HST: None
Number of features	Uniform(5,17)	LR: 5; DT: 8; SVM: 15; RF: 16; XGB: 15, MLP: 16, FIGS: 10, HST: 9
Logistic Regression		
Penalty	l2, l1, elasticnet	elasticnet
C	Uniform(0.5, 2)	0.6662249852612048
Solver	SAGA	SAGA
l1 Ratio	Uniform(0,1)	0.31856895245132366
Decision Tree		
Criterion	gini, entropy	gini
Splitter	best, random	best
Max. Depth	Uniform(3,5)	3
SVM		
Kernel	linear, rbf, sigmoid, poly	rbf
C	Uniform(0,1)	0.5096243767199001
Degree	Uniform(2,10)	NA
Gamma	auto, scale	scale
Random Forest		
Num. Estimators	Uniform(10,1000)	787
Criterion	gini, entropy	gini
Max. Depth	Uniform(1,100)	4
Max. Features	sqrt, log2	log2
XGBoost		
Eta	Uniform(0.01, 0.25)	0.20057413447736855
Gamma	Uniform(0,100)	1.3948395933415347
Subsample	Uniform(0.5, 1)	0.75
Lambda	Uniform(0, 5)	2.6673284087546447
Alpha	Uniform(0,5)	1.6265515257949819
Num. Estimators	Uniform(10,1000)	918
Max. Depth	Uniform(1,100)	4
Scale Pos. Weight	Uniform(0,100)	88.29
Multi-layer Perceptron		
Activation	relu, logistic, tanh	logistic
Solver	adam, lbfgs, sgd	lbfgs
Alpha	Uniform(0.0001, 0.1)	0.05103420653681868
Learning rate	constant, adaptive, invscaling	NA
Beta_1_	Uniform(0,1)	NA
Beta_2_	Uniform(0,1)	NA
Early stopping	True, False	True
Hidden layer sizes	Uniform(18,100000)	100

After training and hyper-parameter optimization the models were evaluated on the separate internal validation test set in terms of different evaluation metrics, namely: accuracy, sensitivity, specificity, PPV, NPV, Area under the ROC curve (AUC), F1-score (F1), Matthew’s correlation coefficient [31] (MCC) and balanced accuracy, as measures of error rate; Brier score as a measure of calibration; and the standardized Net Benefit (sNB), as a measure of utility.

As we mentioned in the Introduction section, to enhance the ability of the developed ML models to reliably detect control loss conditions, we also developed *cautious prediction* models based on the above mentioned ML models. More specifically, the models developed during the training phase were also considered as cautious prediction models that could abstain whenever the prediction for a given instance to be classified was not sufficiently confident [32]. To this purpose, we considered the confidence scores returned by the models, which were tresholded at a 0.75 cutoff: that is, whenever the confidence score assigned to the predicted label was lower than 0.75, the model was considered as *abstaining* from providing a support. We decided to adopt this cautious prediction approach, rather than alternative techniques such as conformal prediction [33] or three-way decision [34], due to its increased efficiency (the computational complexity cost of the thresholding strategy is *O*(1), while it is on the order of $O(\log n)$, for *n* being the dataset size, for conformal prediction, and $O(2^{|Y|})$, for *Y* being the set of possible labels, for three-way decision), ease of interpretation and also due to its equivalence, in the binary classification setting and under weak assumptions, with the above two mentioned methods [34]. We then evaluated these cautious prediction models according to so-called High-Confidence (HC) evaluation metrics (i.e., metrics that only consider the non-abstained on instances), namely the accuracy, sensitivity, specificity, PPV and NPV, as well as the coverage (i.e., the rate of non-abstained instances over the total number of instances in the test set).

After training and internal validation we also evaluated the generalizability and robustness of the developed models by means of an external validation [35]. Specifically we performed a temporal external validation, through which we evaluated the trained models on a set of data collected at the OGSA institute, as for the internal development set, but in a different time period. The dataset encompassed a total of 1589 individual patient records, collected over the period from January 2021 to October 2023, and the same features as for the internal development set. The distribution of features for the external validation dataset is detailed in Table 3. External validation was performed by evaluating the already trained ML models, including the cautious prediction models, on the external validation dataset in terms of the same metrics considered for the internal validation. We also evaluated the similarity between the internal development set and the external validation set in terms of the degree of correspondence $\Phi$ [35], as a comprehensive measure of the differences between the two settings.

**Table 3:** Table of Descriptive Statistics for Data Features with *P*-Value Analysis for the External Validation dataset: This table presents the descriptive statistics for each feature, comparing the external validation and internal development datasets

Feature	Mean (Ext.)	St.Dev (Ext.)	Mean (Int.)	St.Dev (Int.)	Missing (Ext.)	Missing (Int.)	*P*-value
Age	68.809	10.867	66.720	10.546	0%	0%	< 0.001*
VAS (Preop)	7.229	2.187	7.125	2.123	0.1%	1.2%	0.085
SF12 Physical (Preop)	32.098	7.719	33.024	7.878	0%	0%	0.004*
SF12 Mental (Preop)	49.669	12.552	50.917	11.859	0%	0%	0.028*
EQ5D (Preop)	0.704	0.121	0.722	0.117	0.3%	0.2%	0.001*
Height	166.114	9.080	167.440	9.048	0.8%	1%	0.001*
Weight	75.795	15.432	77.033	15.212	0.8%	1%	0.085
BMI	27.387	4.719	27.387	4.455	0.8%	1%	0.916
Hemoglobin (Preop)	13.832	1.408	14.027	1.387	0.4%	0.5%	0.002*
Feature	Categories (Ext.)			Categories (Int.)			*P*-value
Sex	Female (39.6%), Male (60.4%)			Female (46.7%), Male (53.3%)			0.001*
Hip/Knee	Hip (57%), Knee (43%)			Hip (57%), Knee (43%)			0.800
Intervention	First intervention (91.8%), Revision (8.2%)			First Intervention (96.2%), Revision (2.8%)			< 0.001*
ASA	1 (11.2%), 2(82.0%), 3 (6.8%)			1 (13.6%), 2 (82.8%), 3 (3.6%)			1 (0.095), 2 (0.547), 3 (0.001*)

For all features we evaluated the presence of differences with respect to the internal development dataset. For continuous and ordinal features, differences were assessed using the Mann-Whitney U test, while for categorical features, the Fisher’s exact test was utilized to determine statistical significance

Asterisk denotes a significant difference between the two cohorts, at the 95% confidence level

All software was implemented in Python (v. 3.10.9) using the libraries numpy (v. 1.23.5), scipy (v. 1.9.3), pandas (v. 1.5.2), scikit-learn (v. 1.1.2), imodels (v. 1.4.1), shap (v. 0.41.0), xgboost (v. 1.5.1), matplotlib (v. 3.6.2) and seaborn (v. 0.12.2). The reporting of the methods and results follows the IJMEDI/ChAMAI checklist.

## Results

**Fig. 1:**
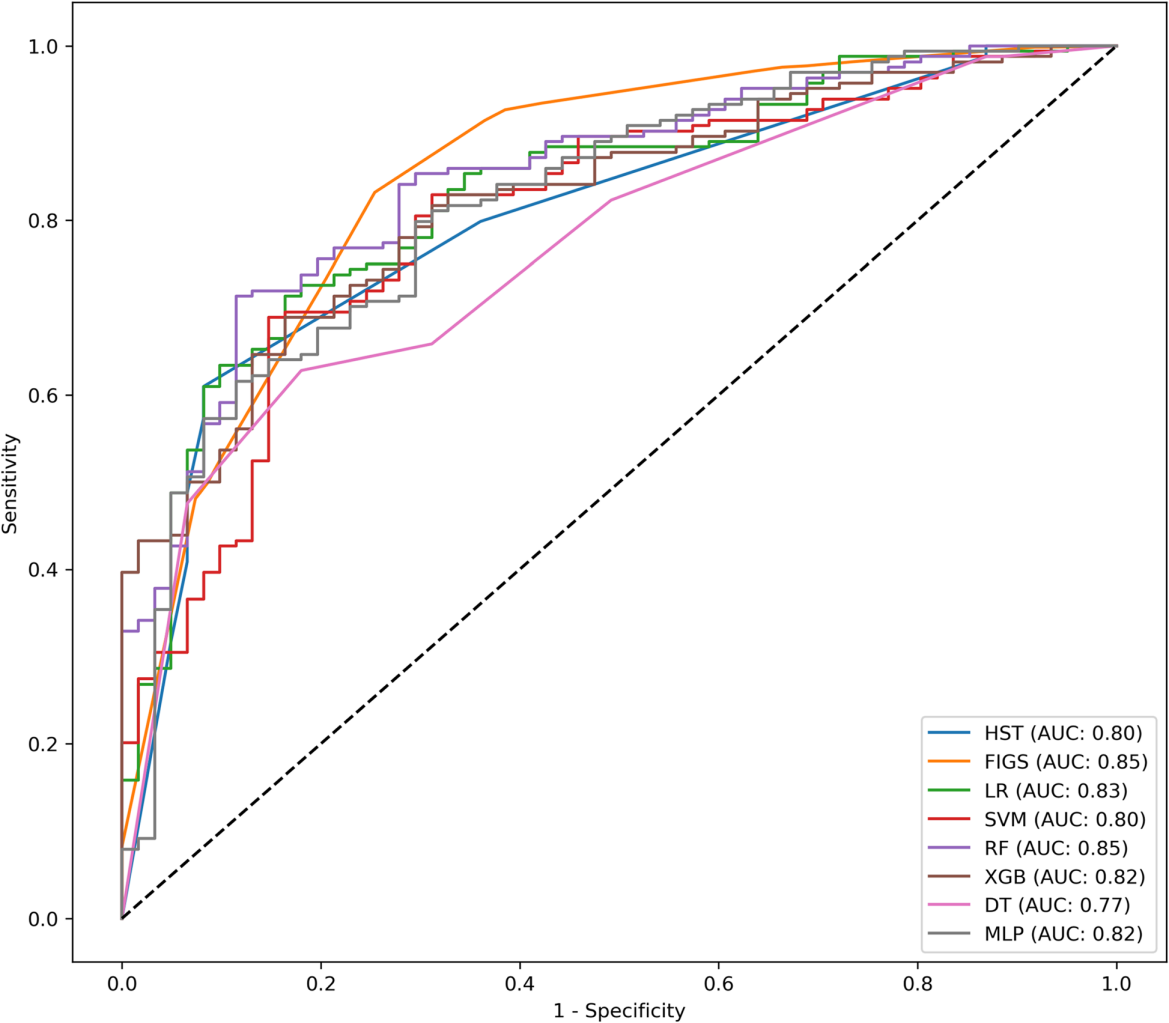
ROC Curves comparing the performance of all developed models. This graph provides a visual comparison of the Receiver Operating Characteristic (ROC) curves for each classifier model established in our analysis: Hierarchical Shrinked Trees (HST), Fast Interpretable Greedy Sums (FIGS), Decision Trees (DT), Random Forest (RF), K-Nearest Neighbors (KNN), Logistic Regression (LR), eXtreme Gradient Boosting (XGB), Support Vector Machines (SVM) and Multi-layer Perceptron (MLP). Each line traces the trade-off between sensitivity (true positive rate) and 1-specificity (false positive rate) across different thresholds. The Area Under the ROC Curve (AUC) is provided for each model as a measure of overall performance

**Fig. 2:**
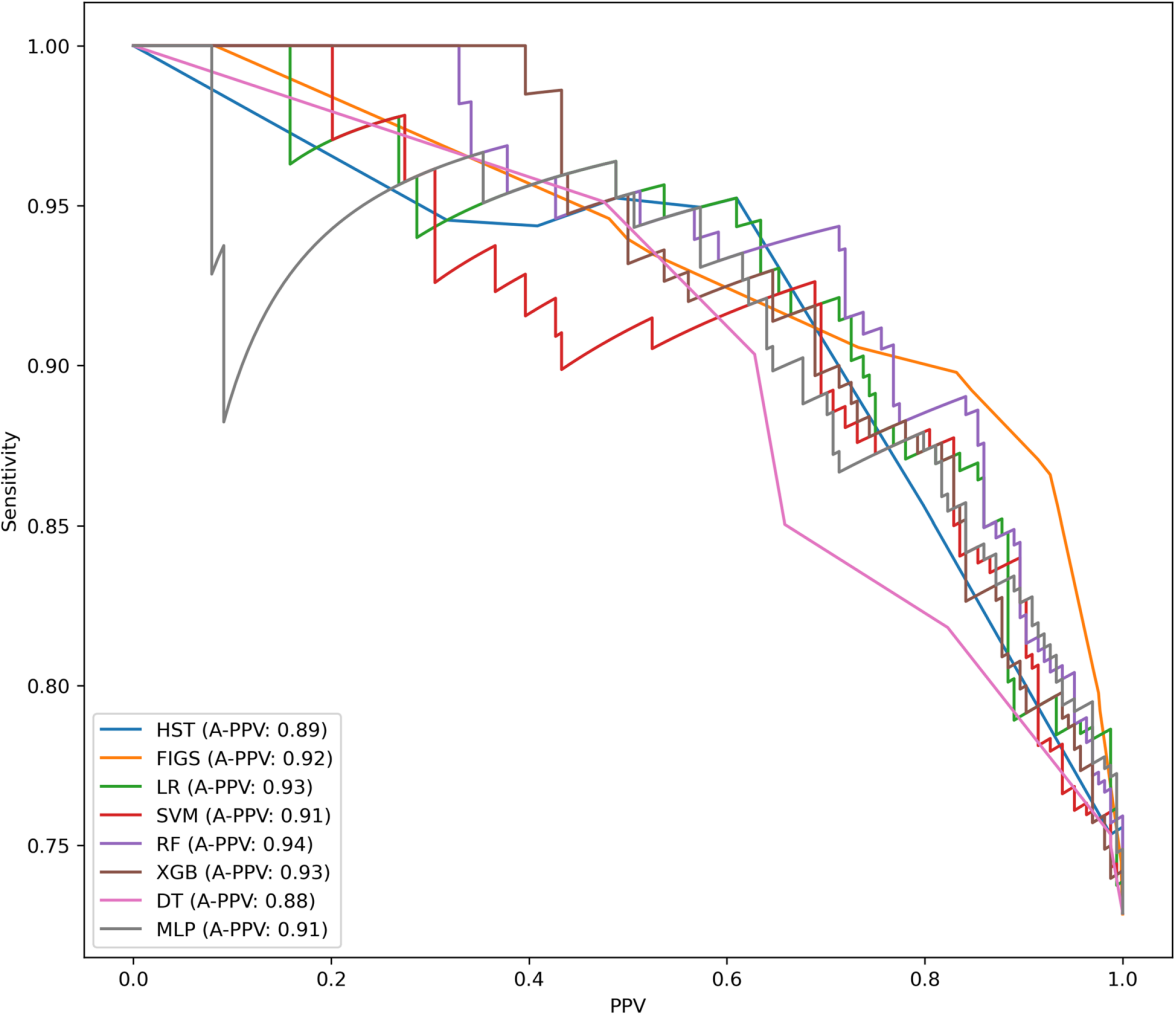
PPV-Sensitivity Curves comparing the performance of all developed models. This graph provides a visual comparison of the PPV-Sensitivity curves for each classifier developed in our analysis: Hierarchical Shrinked Trees (HST), Fast Interpretable Greedy Sums (FIGS), Decision Trees (DT), Random Forest (RF), K-Nearest Neighbors (KNN), Logistic Regression (LR), eXtreme Gradient Boosting (XGB), Support Vector Machines (SVM) and Multi-layer Perceptron (MLP). Each line traces the trade-off between sensitivity (true positive rate) and PPV (positive predictive false) across different thresholds. The Area Under the Precision-Recall (or Positive Predictive Value and Sensitivity) Curve (AUPRC) is provided for each as a measure of overall performance

**Fig. 3:**
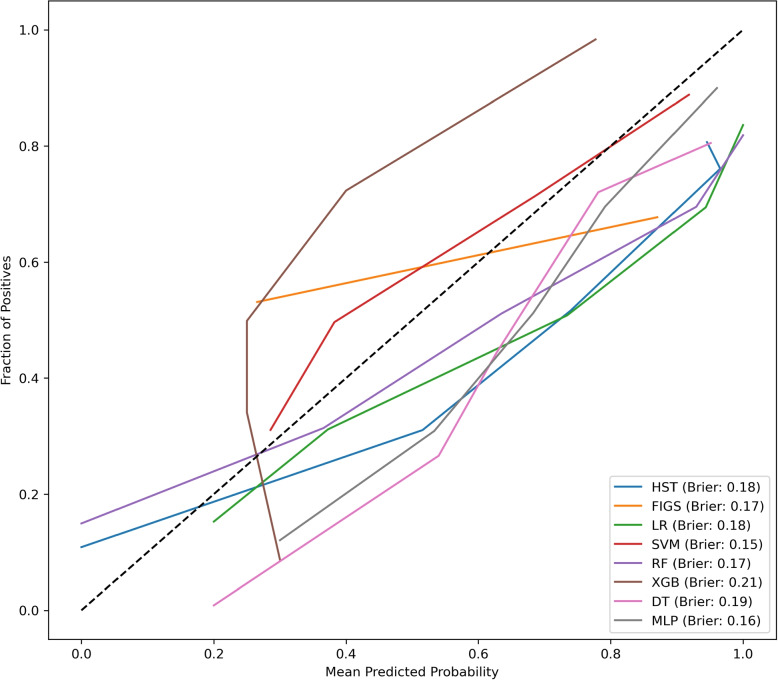
Calibration curves comparing the performance of all developed models. This graph provides a visual comparison of the calibration curves for each classifier developed in our analysis: Hierarchical Shrinked Trees (HST), Fast Interpretable Greedy Sums (FIGS), Decision Trees (DT), Random Forest (RF), K-Nearest Neighbors (KNN), Logistic Regression (LR), eXtreme Gradient Boosting (XGB), Support Vector Machines (SVM) and Multi-layer Perceptron (MLP). Each line represents a model’s ability to estimate the probability of patient improvement after Fast Track (FT) surgery. The closer a curve follows the dashed line (which represents perfect calibration), the more accurately the model predicts the true outcomes. The Brier scores is provided for each as a measure of overall performance, with lower values corresponding to better performance

The results of the developed models are detailed in Table 4 and illustrated in Figs. 1, 2 and 3. The FIGS model emerged as the most effective among the considered models: indeed, for all the considered metrics, except sensitivity and specificity, the performance of FIGS was not significantly lower than that of the top-ranked model. In particular, FIGS was significantly better than all other models in terms of balanced accuracy, AUC and standardized Net Benefit (sNB). By contrast, XGB was the best model in terms of sensitivity, while HST and LR were the best models in terms of specificity: in both cases, FIGS ranked as the second best model. Also when considering the cautious prediction versions of the models, FIGS was among the most effective models, being among the top-ranked models for all considered metrics except sensitivity, and having the best coverage.

**Table 4:** The results of the developed Machine Learning (ML) models are presented along with their respective 95% confidence intervals (C.I.)

	HST	FIGS	LR	SVM	RF	XGB	DT	MLP
Accuracy	0.693 ± 0.06	**0.814** $\varvec{\pm }$ **0.025**	**0.751** $\varvec{\pm }$ **0.056**	**0.742** $\varvec{\pm }$ **0.057**	**0.778** $\varvec{\pm }$ **0.054**	**0.76** $\varvec{\pm }$ **0.056**	0.667 ± 0.062	**0.773** $\varvec{\pm }$ **0.055**
Sensitivity	0.61 ± 0.075	0.847 ± 0.028	0.744 ± 0.067	0.756 ± 0.066	0.799 ± 0.061	**0.97** $\varvec{\pm }$ **0.026**	0.659 ± 0.073	0.799 ± 0.061
Specificity	**0.918** $\varvec{\pm }$ **0.069**	0.725 ± 0.056	**0.77** $\varvec{\pm }$ **0.106**	0.705 ± 0.114	0.721 ± 0.113	0.197 ± 0.1	0.689 ± 0.116	0.705 ± 0.114
PPV	**0.952** $\varvec{\pm }$ **0.041**	**0.892** $\varvec{\pm }$ **0.024**	**0.897** $\varvec{\pm }$ **0.051**	**0.873** $\varvec{\pm }$ **0.055**	**0.885** $\varvec{\pm }$ **0.051**	0.764 ± 0.058	**0.85** $\varvec{\pm }$ **0.062**	**0.879** $\varvec{\pm }$ **0.052**
NPV	**0.467** $\varvec{\pm }$ **0.089**	**0.639** $\varvec{\pm }$ **0.057**	**0.528** $\varvec{\pm }$ **0.104**	**0.518** $\varvec{\pm }$ **0.107**	**0.571** $\varvec{\pm }$ **0.111**	**0.706** $\varvec{\pm }$ **0.217**	**0.429** $\varvec{\pm }$ **0.098**	**0.566** $\varvec{\pm }$ **0.111**
AUC	0.804 ± 0.002	**0.852** $\varvec{\pm }$ **0.001**	0.831 ± 0.002	0.805 ± 0.002	0.848 ± 0.002	0.824 ± 0.002	0.769 ± 0.002	0.82 ± 0.002
F1	0.743 ± 0.058	**0.869** $\varvec{\pm }$ **0.02**	**0.813** $\varvec{\pm }$ **0.048**	**0.81** $\varvec{\pm }$ **0.048**	**0.84** $\varvec{\pm }$ **0.044**	**0.855** $\varvec{\pm }$ **0.038**	0.742 ± 0.056	**0.837** $\varvec{\pm }$ **0.044**
Brier	**0.183** $\varvec{\pm }$ **0.053**	**0.173** $\varvec{\pm }$ **0.024**	**0.183** $\varvec{\pm }$ **0.037**	**0.152** $\varvec{\pm }$ **0.08**	**0.17** $\varvec{\pm }$ **0.028**	**0.213** $\varvec{\pm }$ **0.283**	**0.193** $\varvec{\pm }$ **0.083**	**0.163** $\varvec{\pm }$ **0.083**
Bal. Acc	0.764 ± 0.002	**0.786** $\varvec{\pm }$ **0.001**	0.757 ± 0.002	0.731 ± 0.002	0.76 ± 0.002	0.583 ± 0.001	0.674 ± 0.002	0.752 ± 0.002
MCC	**0.47** $\varvec{\pm }$ **0.128**	**0.552** $\varvec{\pm }$ **0.064**	**0.468** $\varvec{\pm }$ **0.128**	**0.425** $\varvec{\pm }$ **0.128**	**0.487** $\varvec{\pm }$ **0.128**	0.28 ± 0.128	0.311 ± 0.128	**0.473** $\varvec{\pm }$ **0.128**
sNB	0.579 ± 0.003	**0.745** $\varvec{\pm }$ **0.001**	0.659 ± 0.003	0.646 ± 0.004	0.695 ± 0.003	0.671 ± 0.005	0.543 ± 0.004	0.689 ± 0.003
HC Acc.	**0.741** $\varvec{\pm }$ **0.057**	**0.807** $\varvec{\pm }$ **0.026**	**0.789** $\varvec{\pm }$ **0.053**	**0.763** $\varvec{\pm }$ **0.056**	**0.807** $\varvec{\pm }$ **0.052**	**0.781** $\varvec{\pm }$ **0.054**	**0.768** $\varvec{\pm }$ **0.055**	**0.781** $\varvec{\pm }$ **0.054**
HC Sens.	0.67 ± 0.072	0.759 ± 0.033	0.72 ± 0.069	0.697 ± 0.07	0.727 ± 0.068	**0.939** $\varvec{\pm }$ **0.037**	0.729 ± 0.068	0.705 ± 0.07
HC Spec.	**0.907** $\varvec{\pm }$ **0.073**	**0.908** $\varvec{\pm }$ **0.036**	**0.923** $\varvec{\pm }$ **0.067**	**0.895** $\varvec{\pm }$ **0.077**	**0.973** $\varvec{\pm }$ **0.041**	0.375 ± 0.121	**0.886** $\varvec{\pm }$ **0.08**	**0.944** $\varvec{\pm }$ **0.057**
HC PPV	**0.944** $\varvec{\pm }$ **0.044**	**0.946** $\varvec{\pm }$ **0.018**	**0.947** $\varvec{\pm }$ **0.038**	**0.93** $\varvec{\pm }$ **0.042**	**0.982** $\varvec{\pm }$ **0.021**	0.794 ± 0.055	**0.951** $\varvec{\pm }$ **0.037**	**0.965** $\varvec{\pm }$ **0.03**
HC NPV	**0.542** $\varvec{\pm }$ **0.089**	**0.639** $\varvec{\pm }$ **0.057**	**0.632** $\varvec{\pm }$ **0.1**	**0.596** $\varvec{\pm }$ **0.106**	**0.632** $\varvec{\pm }$ **0.108**	**0.706** $\varvec{\pm }$ **0.217**	**0.517** $\varvec{\pm }$ **0.099**	**0.596** $\varvec{\pm }$ **0.11**
Coverage	**0.636** $\varvec{\pm }$ **0.063**	**0.679** $\varvec{\pm }$ **0.031**	0.507 ± 0.065	0.507 ± 0.065	0.507 ± 0.065	0.507 ± 0.065	**0.631** $\varvec{\pm }$ **0.063**	0.507 ± 0.065

These confidence intervals for key metrics such as accuracy, sensitivity, specificity, positive predictive value (*PPV*), and negative predictive value (*NPV*) are calculated based on the variance formula applicable to binomial distributions. In particular, C.I. for the AUC and sNB were computed according to the formulas described in [36]; C.I. for the balanced accuracy were computed according to the formula described in [37]; C.I. for the Brier score were computed according to the formula described in [38]; while C.I. for the MCC were computed by applying Hoeffding’s inequality. For each metric, values in bold denote values that were not significantly worse than the top-ranked one, as measured by overlap of the 95% C.I

**Fig. 4:**
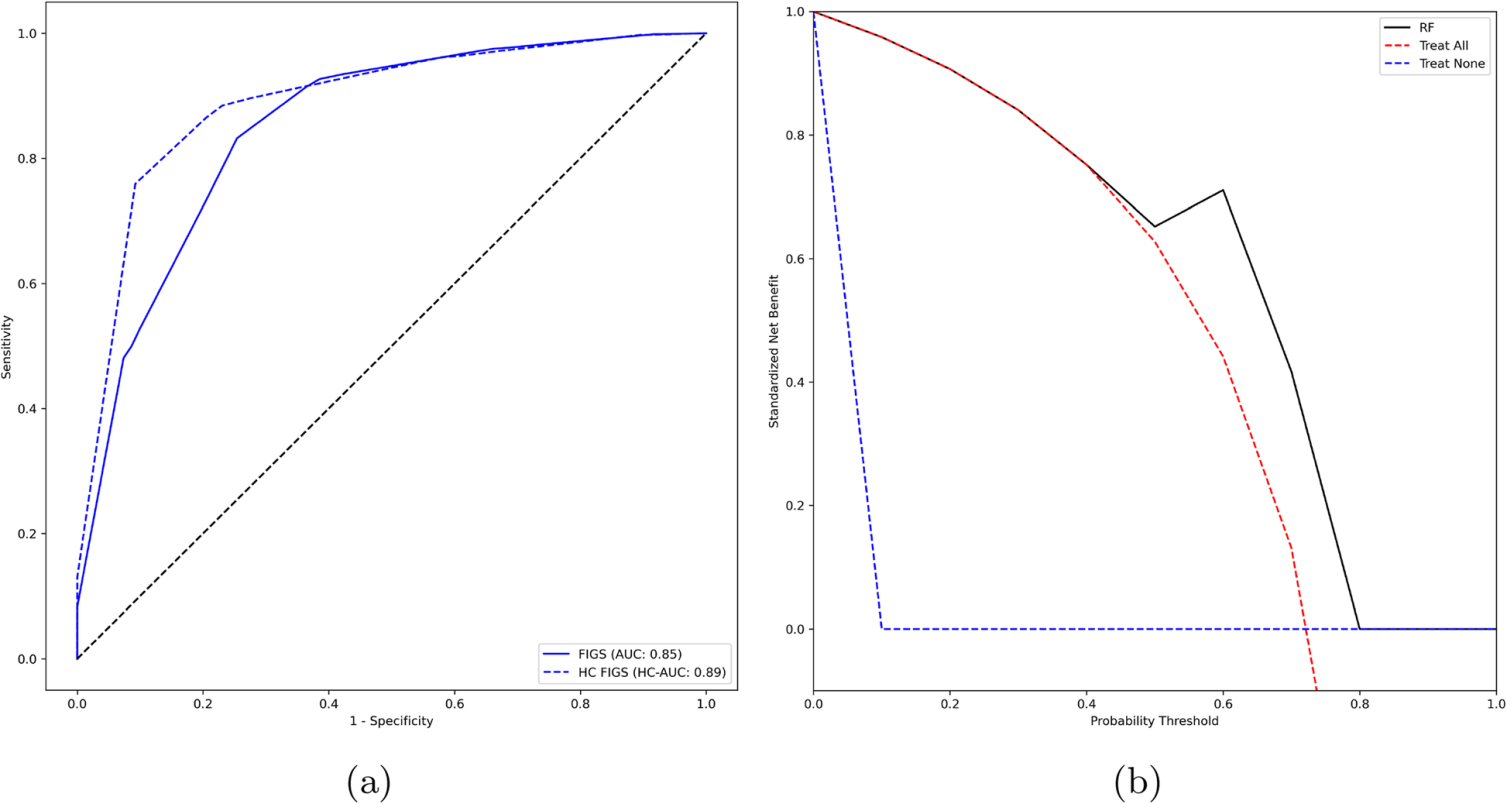
ROC curves (**a**) and Standardized Net Benefit curves (**b**) for the Fast Interpretable Greedy Sums (FIGS). In the ROC curve diagram, the dashed line represents the cautious prediction model based on FIGS. In the Standardized Net Benefit curve diagram, the blue and red dashed lines represent, respectively, the Treat None and Treat All baselines

The results for the best model (FIGS), in terms of both ROC curve (also considering the ROC curve for the corresponding cautious prediction models) and decision curve, are reported in Fig. 4a and b. The FIGS model was uniformly better than the treat-all and treat-none baselines across all probability thresholds. Furthermore, the cautious prediction model based on FIGS improved on the performance of the traditional model across all operating points, and especially so for operating points associated with high specificity (see Fig. 4a).

**Fig. 5:**
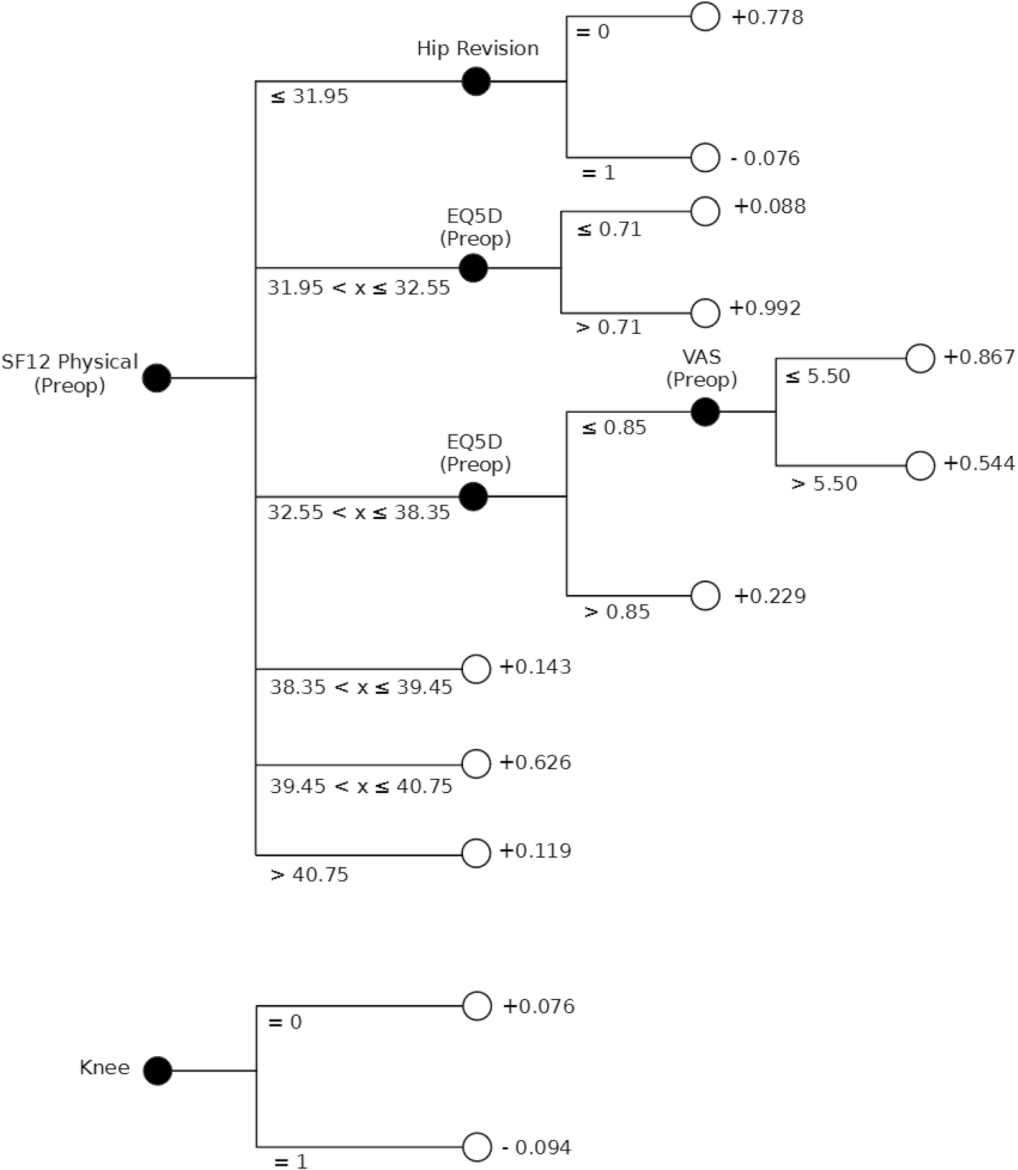
Fast Interpretable Greedy Sums model. The model is represented as a forest of trees that are combined in additive composition. Given an instance *x*, the corresponding probability value for the positive class (in our study, Improved, interpreted as a proxy indicator to confirm assignment to Fast Track) is obtained by following a path in each tree, corresponding to the values of the features of *x*, computing the sum *f*(*x*) of the values associated with the leaves and then applying the sigmoid function $P(y=1 | x) = \sigma \left( f(x)\right) = \frac{1}{1 + e^{-f(x)}}$

**Fig. 6:**
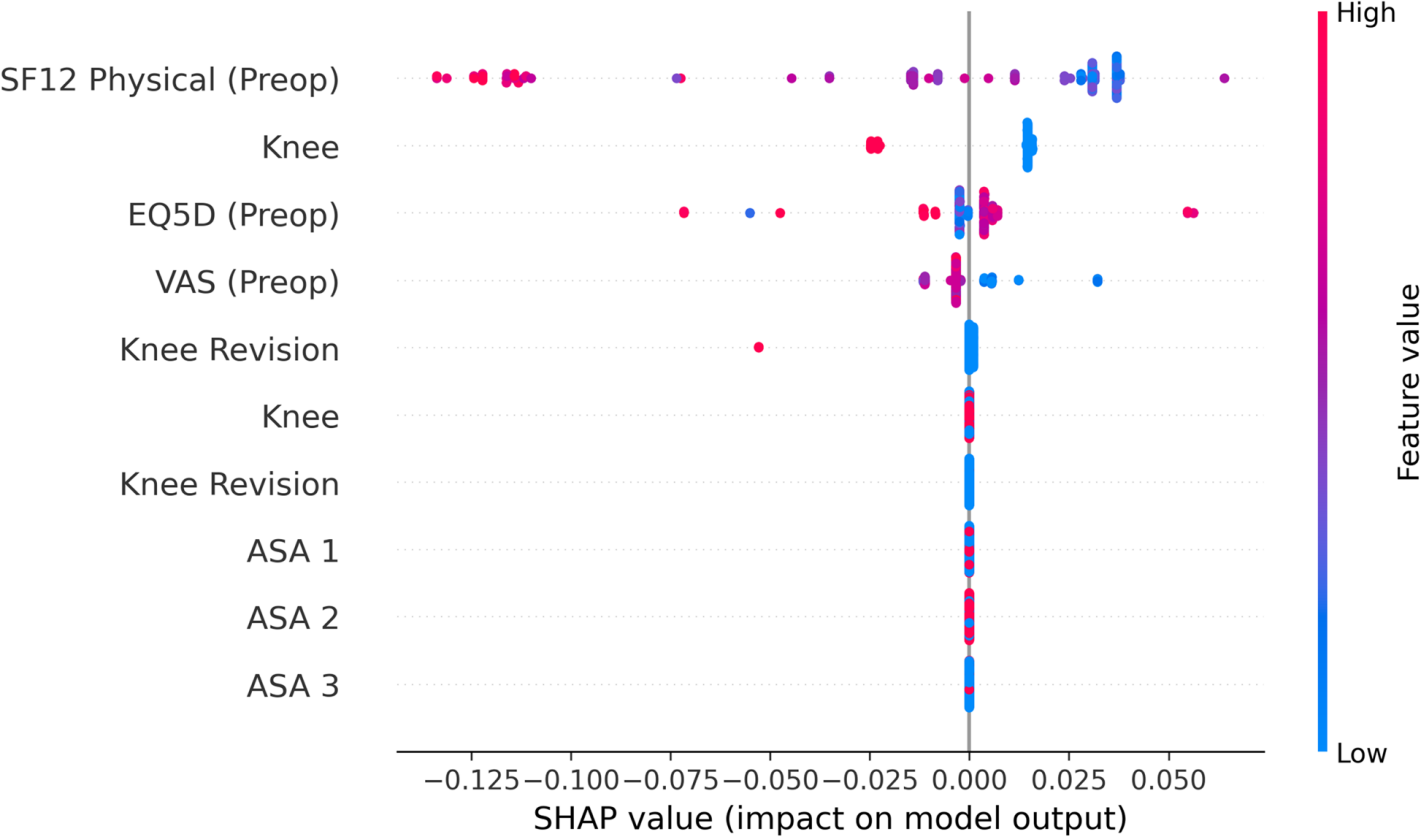
Feature importance for the Fast Interpretable Greedy Sums model, represented in terms of Shapley values. For each feature, the color denotes the magnitude of the features’ values: red denotes high values, while blue denotes low values. Values at the right of the black vertical bar denote increased confidence score for the positive class, while values at the left of the bar denote a corresponding decreased confidence score

So as to provide an additional form of support, according to the tenets of XAI, the FIGS model is depicted in Fig. 5. The FIGS model identified the pre-operative SF12 physical score and the surgical procedure location (knee/hip) as the most predictive features. The same information is confirmed also by the analysis of Shapley values (performed through the SHAP library), shown in Fig. 6, that similarly identified the SF12 physical score and the procedure location as the most important features, followed by the pre-operative EQ5D and VAS which were also considered as highly predictive in the tree representation shown in Fig. 5.

As seen in Tables 1 and 3, the internal development dataset and the external validation dataset significantly differed for most of the continuous features: in particular, the two populations had significantly different distributions in terms of age, SF12 Physical and Mental scores, EQ5D score, height and preoperative hemoglobin. The two populations also differed significantly in terms of the frequency of first interventions and revisions in the knee arthroplasty sub-cohort. The overall similarity between the two dataset was $\Phi = 0.5$, which, according to the scale defined in [35], corresponds to a *moderate* level of similarity. The results of the external validation are reported in Table 5. The FIGS model performance significantly worsened w.r.t. balanced accuracy, AUC and sNB: however, for all of these metrics, as well as for accuracy, F1 score and Brier score, the performance of FIGS was not significantly worse than that of the best performing model, and were always higher than 0.70. In particular, FIGS was the model with the highest balanced accuracy, AUC and sNB: for these last two metrics, the performance of FIGS was significantly better than for all other models. In terms of cautious prediction models, no model significantly worsened as compared to the internal validation, with the exception of the XGB and MLP models for the HC PPV metric.

**Table 5:** The results of the developed Machine Learning (ML) models on the external validation dataset are presented along with their respective 95% confidence intervals (C.I.)

	HST	FIGS	LR	SVM	RF	XGB	DT	MLP
Accuracy	0.687 ± 0.061	**0.784** $\varvec{\pm }$**0.054**	**0.734** $\varvec{\pm }$**0.058**	**0.751** $\varvec{\pm }$**0.057**	**0.800** $\varvec{\pm }$**0.052**	**0.798** $\varvec{\pm }$**0.053**	**0.712** $\varvec{\pm }$**0.059**	0.724 ± 0.022
Sensitivity	0.623 ± 0.037	0.820 ± 0.029	0.741 ± 0.033	0.756 ± 0.033	0.811 ± 0.029	**0.994** $\varvec{\pm }$**0.006**	0.695 ± 0.035	0.750 ± 0.025
Specificity	**0.866** $\varvec{\pm }$**0.043**	0.716 ± 0.059	0.715 ± 0.057	0.736 ± 0.056	0.721 ± 0.056	0.255 ± 0.055	0.757 ± 0.054	0.658 ± 0.044
Balanced accuracy	0.744 ± 0.000*	**0.768** $\varvec{\pm }$0.001*	0.728 ± 0.001*	0.746 ± 0.001	**0.766** $\varvec{\pm }$**0.001**	0.625 ± 0.001	0.726 ± 0.001	0.704 ± 0.001*
PPV	**0.928** $\varvec{\pm }$**0.024**	0.878 ± 0.026	0.878 ± 0.027	0.888 ± 0.026	0.897 ± 0.024	0.787 ± 0.028	0.888 ± 0.027	0.847 ± 0.022
NPV	0.454 ± 0.046	0.580 ± 0.058	0.500 ± 0.053	0.522 ± 0.053	0.600 ± 0.056	**0.938** $\varvec{\pm }$**0.058**	0.474 ± 0.050	0.511 ± 0.041
AUC	0.812 ± 0.001	**0.813** $\varvec{\pm }$0.001*	0.811 ± 0.001*	0.809 ± 0.001	0.812 ± 0.000*	0.807 ± 0.000*	0.794 ± 0.001	0.759 ± 0.001*
F1	0.745 ± 0.029	**0.848** $\varvec{\pm }$**0.021**	0.804 ± 0.024	**0.817** $\varvec{\pm }$**0.024**	**0.858** $\varvec{\pm }$**0.021**	**0.851** $\varvec{\pm }$**0.018**	0.780 ± 0.026	0.796 ± 0.019
Brier	**0.176** $\varvec{\pm }$**0.052**	**0.177** $\varvec{\pm }$**0.026**	**0.184** $\varvec{\pm }$**0.041**	**0.145** $\varvec{\pm }$**0.076**	**0.161** $\varvec{\pm }$**0.027**	**0.170** $\varvec{\pm }$**0.024**	**0.180** $\varvec{\pm }$**0.075**	**0.193** $\varvec{\pm }$**0.12**
sNB	0.574 ± 0.001*	**0.728** $\varvec{\pm }$0.000*	0.638 ± 0.001*	0.661 ± 0.001	0.727 ± 0.001	0.724 ± 0.001	0.608 ± 0.001	0.615 ± 0.001*
MCC	**0.352** $\varvec{\pm }$**0.049**	**0.391** $\varvec{\pm }$**0.049**	**0.372** $\varvec{\pm }$**0.049**	**0.365** $\varvec{\pm }$**0.049**	**0.426** $\varvec{\pm }$**0.049**	**0.350** $\varvec{\pm }$**0.049**	0.328 ± 0.049	**0.383** $\varvec{\pm }$**0.049**
HC accuracy	**0.729** $\varvec{\pm }$**0.058**	**0.772** $\varvec{\pm }$**0.055**	**0.771** $\varvec{\pm }$**0.055**	**0.776** $\varvec{\pm }$**0.055**	**0.820** $\varvec{\pm }$**0.050**	**0.722** $\varvec{\pm }$**0.059**	**0.785** $\varvec{\pm }$**0.054**	0.740 ± 0.022
HC sensitivity	0.631 ± 0.037	0.716 ± 0.034	0.697 ± 0.035	0.701 ± 0.035	0.742 ± 0.033	**0.985** $\varvec{\pm }$**0.009**	0.759 ± 0.033	0.684 ± 0.027
HC specificity	**0.944** $\varvec{\pm }$**0.029**	**0.901** $\varvec{\pm }$**0.048**	**0.936** $\varvec{\pm }$**0.031**	**0.937** $\varvec{\pm }$**0.031**	**0.964** $\varvec{\pm }$**0.020**	0.335 ± 0.060	0.854 ± 0.045	0.846 ± 0.034*
HC PPV	**0.961** $\varvec{\pm }$**0.018**	**0.963** $\varvec{\pm }$**0.018**	**0.960** $\varvec{\pm }$**0.016**	**0.960** $\varvec{\pm }$**0.016**	**0.982** $\varvec{\pm }$**0.011**	0.686 ± 0.032*	0.934 ± 0.021	0.893 ± 0.019*
HC NPV	0.539 ± 0.046	0.580 ± 0.058	0.582 ± 0.052	0.591 ± 0.052	0.658 ± 0.054	**0.938** $\varvec{\pm }$**0.058**	0.565 ± 0.050	0.587 ± 0.04
Coverage	**0.567** $\varvec{\pm }$**0.065**	**0.669** $\varvec{\pm }$**0.062**	0.501 ± 0.065	0.501 ± 0.065	0.501 ± 0.065	0.501 ± 0.065	**0.651** $\varvec{\pm }$**0.062**	0.501 ± 0.025

For each metric and and model, an asterisk (*) denotes that the performance of that model on the external validation dataset was significantly worse than on the internal validation dataset

Asterisk denotes a significant difference between the two cohorts, at the 95% confidence level

## Discussion

In recent years, the incidence of hip and knee arthroplasties has steadily increased [39, 40], due to an increasingly aging population. Such treatment procedures, while providing benefits in terms of life quality to the treated patients [41], may have a complex rehabiliation and follow-up as well as have a significant impact on national health systems. For this reason, FT protocols have become especially pertinent in managing such surgical procedures, to reduce hospital stays and associated costs, as well as for improving patients’ satisfaction and perceived life quality [20, 42–44]. Despite these benefits, however, the criteria for assigning patients to FT are still mostly based around qualitative assessments formulated by the managing clinician that do not comprehensively take into account patients’ data [45, 46], including PROMs.

Our study has contributed to this field by demonstrating for the first time in the literature, up to our knowledge, the effective application of ML as a way to develop second-opinion decision support systems to optimize the assignment of patients to FT surgical protocols for these orthopedic surgeries. As healthcare systems grapple with the demands of an aging population [47], our approach to enhancing decision-making in patient assignment to FT procedures fills a critical gap, by providing clinicians with a quantitative tool that helps them validate and optimize the protocol assignment decisions they have formulated for any given patient. To do so, the developed ML models leverage the extensive patient data available, including PROMs (that were identified as being among the most important predictive feature, see Figs. 5 and 6), thus addressing a previously underutilized resource in patient care optimization [48].

To more effectively leverage the use of ML models as second-opinion support systems, the core of our contribution lies in the incorporation of controllable AI principles [24], particularly XAI and cautious prediction, in the development of such models, so as to align with the need for accountability and transparency in AI applications in healthcare [49, 50]. Indeed, we showed that interpretable models, and particularly so the FIGS model, have performance on par or even better than the best black-box model we considered (i.e., Random Forest), indicating that accuracy does not have to be sacrificed for interpretability [51]. Achieving high sensitivity, specificity, and PPV, along with an AUC greater than 80%, this model underscores the viability of ML and PROMs in predicting whether a patient, preliminarily assigned to FT by the managing clinicians, will have favorable post-surgery outcomes: such an indication is used as proxy for the actual effectiveness of the protocol assignment decision formulated by the clinician, and can thus be used to either validate this preliminary decision or to notify the doctor that further information should be collected for selecting the optimal rehabilitation protocol for the given patient. Furthermore, the application of cautious prediction further enhanced the performance of the developed models, showing how providing ML models with uncertainty quantification and abstention capabilities can make them more accurate as well as provide the clinicians with an important indication about the reliability of the support they provide. Such an approach not only fosters clinician trust in AI [52] but also ensures that AI supports rather than supplants clinical judgment [53], in perfect agreement with the second-opinion approach.

Finally, our study’s external validation, further testifies to the robustness and generalizability of our models: indeed, while unsurprisingly for some metrics the developed models showed a decrease in performance as compared with the internal validation, their error rates remained well within reasonable quality ranges [54]. Interestingly, it was on the external validation that controllable AI approaches best showed their potential: indeed, in all cases, the performance of the cautious prediction models did not decrease significantly as compared with the internal validation, showing that providing such a form of uncertainty quantification can not only improve reliability and trust, but also generalizability and robustness [52, 55].

Obviously, this study is not without limitation. Firstly, the study being of a retrospective nature, we did not evaluate the effectiveness of the developed ML models in clinical practice: we believe that future prospective studies should evaluate the performance of the developed models when deployed in real-world scenarios [56]. Nonetheless, to this regard, we note that we did not limit our evaluation to an internal validation, but rather also externally validated the developed models. Such an analysis, while not being as informative as a prospective study, provides additional indications about the developed models’ robustness and generalizability [57, 58]. Secondly, and with regard to the external validation previously mentioned, we note that our validation procedure considered data coming from the same institute from which the development data were collected. Thus, while we considered the stability of the developed models to time-related shifts [59], we did not evaluate their transportability to other clinical settings [60]. This is an important consideration [35, 58], as different hospitals may have different criteria for assigning patients to FT or Care-as-Usual protocols, as well as different patients’ populations. Therefore, we believe that multi-centric validation studies would be particularly relevant for confirming (or disproving) the generalizability of the developed models. Finally, in our study, we adopted an approach to ML model development grounded in the principles of controllable AI, with specific reference to providing models that are both explainable as well as able to provide an indication of their predictive uncertainty: we motivated this design choice by highlighting the importance of controllability for the development of second-opinion support systems [49], and specifically so providing such systems with the ability to detect control loss situations and notify them to the managing clinician [61]. While we showed how interpretable and cautious models reported performance on-par with, or even better than, traditional and black-box models, we did not perform any user validation aimed at assessing the actual effectiveness of providing such support to the clinicians [62]. While there have been some recent studies showing how providing domain experts with controllable support could prove more effective for both improving accuracy as well as limiting the risk of emergence of cognitive biases (e.g., automation bias) [62, 63], the research on this topic is still limited: thus, we believe this to be a particularly relevant direction for future research, both in terms of analyzing the impact of providing cautious prediction support to clinicians, as well as performing clinical validation of the developed interpretable model (see Fig. 5).

## Conclusions

This article has explored the development of ML models in the context of Fast Track surgical procedures, particularly focusing on hip and knee arthroplasties. Our research underscores the increasing relevance of such predictive models in the current healthcare landscape, which is marked by a growing aging population and the consequent rise in demand for efficient and cost-effective surgical management.

Our study demonstrated that ML algorithms can significantly enhance the process of assigning patients to FT protocols. By accurately predicting the improvement in patients’ health status, these models can be used to offer a reliable second-opinion to support clinical decisions. This not only aids in optimizing patient outcomes but also plays a crucial role in reducing the length of hospital stays and associated costs.

Furthermore, our research highlighted the importance of XAI techniques in making these predictive models more transparent and understandable to clinicians. This aspect of controllable AI ensures that the decision-making process remains in the hands of healthcare professionals, thereby enhancing the reliability and ethical integrity of using AI systems in medical settings. We also showed how cautious prediction, another form of controllable AI, could be used to reliably increase the robustness and uncertainty quantification capabilities of predictive models, enabling the clinicians to make more accurate and more informed decisions.

Thus, the adoption of ML models in the assignment of patients to FT procedures represents a significant stride towards improving the appropriateness of post-surgical care, which requires further research and validation studies. Doing so aims to contribute to the broader goal of making healthcare more sustainable, particularly in the face of challenges posed by an aging population and increased demand for medical services. By leveraging predictive analytics, healthcare systems can not only help physicians get better patient outcomes but also help them manage resources more effectively, paving the way for a more resilient and responsive healthcare system.

## Data Availability

Access to the de-identified data for all the cohorts involved in the study may be made available upon reasonable request to the authors. Access to the computer code used in this research is available upon reasonable request to the authors.

## Abbreviations

AUC: Area under the ROC curve
CP: Cautious prediction
DT: Decision tree
EHR: Electronic health records
FIGS: Fast interpretable greedy-tree sums
FT: Fast track
HC: High-confidence
HST: Hierarchical shrinked trees
LR: Logistic regression
MCID: Minimum clinically important difference
ML: Machine learning
MLP: Multi-layer perceptron
NPV: Negative predictive value
OGSA: IRCCS Ospedale Galeazzi - Sant’Ambrogio
PPV: Positive predictive value
PROM: Patient reported outcome measure
RF: Random forest
sNB: standardized Net Benefit
SVM: Support vector machine
XAI: eXplainable AI
XGB: XGBoost

## Footnotes

1 The MCID for the SF12 Physical score varied according to the primary affected location: 3.68 for knee and 3.80 for hip.
